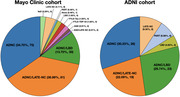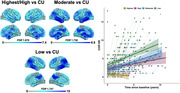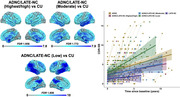# Clinical criteria for a limbic‐predominant amnestic neurodegenerative syndrome highly associated with TDP‐43 and slow clinical progression

**DOI:** 10.1002/alz.084087

**Published:** 2025-01-03

**Authors:** Nick Corriveau‐Lecavalier, Hugo Botha, Jonathan Graff‐Radford, Aaron Switzer, Scott A. Przybelski, Heather J. Wiste, Melissa E. Murray, Ross R. Reichard, Dennis W. Dickson, Aivi T. Nguyen, Vijay K. Ramanan, Stuart J. McCarter, Brad Boeve, Mary M. Machulda, Julie A. Fields, Nikki H. Stricker, Peter T Nelson, Michel J. Grothe, David S. Knopman, Val J. Lowe, Ronald C. Petersen, Clifford R. Jack, David T. Jones

**Affiliations:** ^1^ Mayo Clinic, Rochester, MN USA; ^2^ Department of Neurology, Mayo Clinic, Rochester, MN USA; ^3^ Department of Quantitative Health Sciences, Mayo Clinic, Rochester, MN USA; ^4^ Mayo Clinic, Jacksonville, FL USA; ^5^ Department of Laboratory Medicine and Pathology, Mayo Clinic, Rochester, MN USA; ^6^ Department of Neuroscience, Mayo Clinic, Jacksonville, FL USA; ^7^ Department of Psychiatry and Psychology, Mayo Clinic, Rochester, MN USA; ^8^ College of Medicine, University of Kentucky, Lexington, KY USA; ^9^ German Center for Neurodegenerative Diseases (DZNE), Rostock Germany

## Abstract

**Background:**

Limbic‐predominant age‐related TDP‐43 encephalopathy (LATE) is a neuropathologically‐defined disease, and it is frequently comorbid with Alzheimer’s disease neuropathological change (ADNC). However, the neurological syndrome associated with LATE neuropathological change (LATE‐NC) is not defined. We propose a set of clinical criteria for a limbic‐predominant amnestic neurodegenerative syndrome (LANS) that is highly associated with LATE‐NC. These criteria assess degree of certainty (highest, high, moderate, low) based on features that are measurable *in vivo*, including older age at evaluation, a mild clinical syndrome, impaired semantic knowledge, disproportionate hippocampal atrophy, limbic hypometabolism, absence of neocortical degeneration patterns and low likelihood of neocortical tau. We operationalized these criteria using two large datasets with clinical and pathologic outcomes.

**Method:**

We screened autopsied patients from Mayo Clinic and ADNI cohorts and applied the LANS criteria to those with an antemortem predominant amnestic syndrome (Mayo, *n* = 165; ADNI, *n* = 53). We compared frequencies of neuropathological diagnoses across LANS likelihoods, compared longitudinal CDR‐SB trajectories across LANS likelihoods, and stratified ADNC/LATE‐NC patients according to their LANS likelihood and compared their longitudinal CDR‐SB trajectories to those with ADNC or LATE‐NC.

**Result:**

ADNC, ADNC/LATE‐NC and LATE‐NC accounted for 35%, 37% and 4% of cases in the Mayo cohort, respectively, and 30%, 22%, and 9% of cases in the ADNI cohort, respectively (Fig. 1). ADNC cases had the lowest LANS likelihoods, LATE‐NC patients had the highest likelihoods, and ADNC/LATE‐NC patients had intermediate likelihoods. Patients with high LANS likelihoods had a milder and slower clinical course and more severe temporo‐limbic degeneration compared to those with low likelihoods (Fig. 2). ADNC/LATE‐NC patients with higher likelihoods had more temporo‐limbic degeneration and a slower rate of cognitive decline, and those with lower likelihoods had more lateral temporo‐parietal degeneration and a faster rate of cognitive decline (Fig. 3).

**Conclusion:**

The implementation of LANS criteria has implications for disambiguating the different driving etiologies of progressive amnestic presentations in older age to guide prognosis, treatment, and clinical trial design and enrollment. The development of *in vivo* biomarkers specific to TDP‐43 pathology are needed to further refine molecular associations between LANS and LATE‐NC and precise antemortem diagnoses of LATE.